# ﻿Two new *Pleroma* species and an updated key: Melastomateae from the Serra da Canastra National Park, Minas Gerais, Brazil

**DOI:** 10.3897/phytokeys.247.130040

**Published:** 2024-09-26

**Authors:** Rosana Romero, Rodrigo Pereira Silva, Paulo José Fernandes Guimarães

**Affiliations:** 1 Instituto de Biologia, Universidade Federal de Uberlândia, Rua Ceará, s.n., 38400-902, Uberlândia, Minas Gerais, Brazil Universidade Federal de Uberlândia Uberlândia Brazil; 2 Diretoria de Pesquisa Científica, Instituto de Pesquisas Jardim Botânico do Rio de Janeiro, Rua Pacheco Leão, 915, Rio de Janeiro, RJ, 22460-030, Brazil Instituto de Pesquisas Jardim Botânico do Rio de Janeiro Rio de Janeiro Brazil

**Keywords:** Campo rupestre, endemism, Melastomateae, pedoconnective, *Svitramia*, taxonomy, Campo rupestre, endemismo, Melastomateae, pedoconectivo, *Svitramia*, taxonomia

## Abstract

Here we describe and illustrate *Pleromacanastrense***sp. nov** and *Pleromaviscosa***sp. nov.** two new species of the Melastomateae tribe from Serra da Canastra National Park, Minas Gerais, Brazil. We also provide an updated identification key for the members of the tribe that occur in this Protected Area. *Pleromacanastrense***sp. nov.** has coriaceous leaves, broadly ovate to orbicular leaf blade, entire and adpressed-strigose margin, 11–17 basal acrodromous veins, and flowers with white petals. *Pleromaviscosa***sp. nov.** has the younger branches, both side of the leaf blade, bracteoles, hypanthium, and sepals, densely covered by viscous trichomes, as well as prominent secondary veins on the abaxial surface of the blade. Both species have stamens with a short pedoconnective and an inconspicuous ventral appendage. In addition to the descriptions of new species, we present comments, geographic distribution data, conservation status and images of plants in the field. We recommend that *P.canastrense* and *P.viscosa* should be included as of ‘Least Concern’ (LC) in the IUCN Red List.

## Abstract (pt)

Descrevemos e ilustramos aqui *Pleromacanastrense***sp. nov** e *Pleromaviscosa***sp. nov.**, duas novas espécies da tribo Melastomateae do Parque Nacional da Serra da Canastra, Minas Gerais, Brasil. Fornecemos também uma chave de identificação atualizada para os membros da tribo que ocorrem nesta área protegida. *Pleromacanastrense***sp. nov.** apresenta folhas coriáceas, lâmina foliar amplamente oval a orbicular, margem inteira e estrigosa, 11–17 nervuras acródromas basais e flores com pétalas brancas. Enquanto *Pleromaviscosa***sp. nov.** apresenta os ramos mais jovens, ambas as faces da lâmina foliar, bractéolas, hipanto e sépalas densamente cobertos com tricomas viscosos, além de nervuras secundárias proeminentes na face abaxial da lâmina. As duas espécies apresentam estames com pedoconectivo curto e apêndice ventral inconspícuo. Além das descrições das novas espécies, apresentamos comentários, dados de distribuição geográfica, estado de conservação e imagens das plantas no campo. Recomendamos que *P.canastrense* e *P.viscosa* sejam incluídas na categoria ‘Pouco Preocupante’ na Lista Vermelha da IUCN.

## ﻿Introduction

The Serra da Canastra National Park, located in southwestern Minas Gerais (IBAMA 2005), is considered the second-largest conservation unit in the state (Romero and Martins 2002; Machado and Romero 2014; Romero and Versiane 2014) and the sixth-largest area protected in Brazil (IBAMA 2005). The Park has a diverse flora with 101 families of Angiosperms and at least 17 endemism areas with 45 endemic species (see Romero and Nakajima 1999). However, these numbers must be higher since dozens of new species endemic to the region have been described in the last 30 years (*e.g.*, Versieux and Wanderley 2008; Batista et al. 2010; Chautems et al. 2010; Feres 2010; Romero and Versiane 2014; Oliveira et al. 2016; Romero and Rocha 2017; Oliveira et al. 2019; Echternacht et al. 2021).

Among the most diverse families in this National Park, Melastomataceae Juss. stands out with 105 species in 21 genera (see Romero and Martins 2002; Silva and Romero 2008; Romero and Versiane 2014). The pantropical Melastomateae Bartl. tribe is represented in the National Park by 24 species distributed in *Pleroma* (18 spp.), *Chaetogastra* (3 spp.), and *Macairea*, *Pterolepis*, and *Tibouchina* (with one species each) (see Romero and Martins 2002; Silva and Romero 2008; Romero and Versiane 2014; Alves 2022; Silva 2023).

The phylogenetic study of the tropical specimens of Melastomateae recognized 17 genera (Michelangeli et al. 2013; Veranso-Libalah et al. 2022), and the most significant implication was the segregation of *Tibouchina* Aubl. This genus was previously considered the largest within the tribe, with 310 species (Guimarães et al. 2019). Then, *Tibouchina* was redefined into four monophyletic and easily distinguishable genera (Guimarães et al. 2019). The narrower circumscription of *Tibouchina* led to the reinstatement of *Pleroma* and *Chaetogastra* DC., along with the description of a new genus, *Andesanthus* P.J.F.Guim. & Michelang., with *Pleroma* also including *Itatiaia* Ule, *Microlepis* Schrad. ex Nees, and *Svitramia* Cham.

The six species previously recognized in *Svitramia* occur in the Serra da Canastra National Park: *S.pulchra* Cham. [=*Pleromabandeirae* (Cham.) P.J.F.Guim. & Michelang.], *S.hatschbachii* Wurdack [=*P.gertii* (Wurdack) P.J.F.Guim. & Michelang.], *S.integerrimum* R.Romero & A.B.Martins [=*P.integerrimum* (R.Romero & A.B.Martins) P.J.F.Guim. & Michelang.], *S.minor* R.Romero & A.B.Martins [=*P.minus* (R.Romero & A.B.Martins) P.J.F.Guim. & Michelang.], *S.petiolata* R.Romero & A.B.Martins [=*P.petiolatum* (R.Romero & A.B.Martins) P.J.F.Guim. & Michelang.], and S. *wurdackiana* R.Romero & A.B.Martins (=*P.wurdackianum* (R.Romero & A.B.Martins) P.J.F.Guim. & Michelang.] (see Romero 2000; Romero and Martins 2003). Here, we describe two new species of *Pleroma*, initially referred to as *Svitramia* sp. nov. 1 and *Svitramia* sp. nov. 2 in the previous Melastomataceae checklist for Serra da Canastra National Park (see Romero and Martins 2002). We compare the new species with morphologically similar species and provide illustrative plates, field images, and an occurrence map of the new species. In addition, the identification key for the species of Melastomateae in the Serra da Canastra National Park is provided.

## ﻿Methods

This study was based on the examination of *Chaetogastra*, *Pleroma*, *Pterolepis*, *Macairea*, and *Tibouchina* specimens from Serra da Canastra National Park deposited at the HUFU with duplicates at CESJ, HUEFS, K, NY, OUPR, P, R, RB, SP, SPF, UB, UEC, UFG, VIC herbaria (Thiers 2024, continuously updated) and field observations. We also examined specimens with available images on Global Plants on Jstor (https://plants.jstor.org/), *species*Link (https://specieslink.net/), and Reflora Virtual Herbaria (http://reflora.jbrj.gov.br/reflora/herbarioVirtual). The vegetative and reproductive structures of the examined samples of the new species were measured in a stereoscopic microscope and with a digital caliper. Morphological terminology followed Radford (1986). The area of occupancy (AOO) was estimated using GeoCAT software (Bachman et al. 2011), and preliminary assessments of conservation status followed IUCN criteria (2001, 2022).

## ﻿Results

### ﻿Taxonomic treatment

#### ﻿ Pleroma canastrense

Taxon classificationPlantaeMyrtalesMelastomataceae

R.Romero, R.Pereira & P.J.F.Guim. sp. nov.

67F339E3-92F5-5F0D-8EBE-3C06C080215C

urn:lsid:ipni.org:names:77349019-1

Figs 1 , 2 , 3

##### Type.

Brazil • Minas Gerais. Parque Nacional da Serra da Canastra. São Roque de Minas: campo rupestre do morro próximo à sede administrativa do parque; 26 June 1994; fl. fr., *R. Romero & J.N. Nakajima 1039* (holotype HUFU!; isotypes: BHCB!, RB!, UEC!).

**Figure 1.**
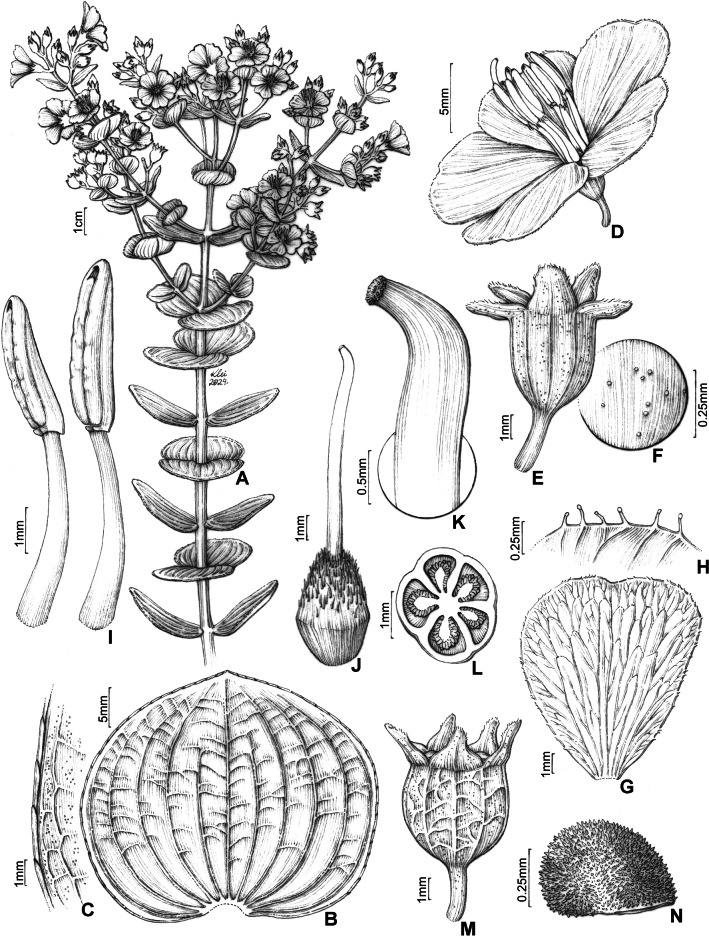
*Pleromacanastrense* R.Romero, R.Pereira & P.J.F.Guim.: **A** flowering branch **B** adaxial leaf surface **C** detail of the leaf blade margin **D** flower **E** hypanthium and sepals **F** detail of the hypanthium surface **G** petal, abaxial view **H** petal margin detail **I** stamens, in lateral view **J** gynoecium **K** stigma detail **L** cross section of the ovary **M** fruit **N** seed (Illustration by Klei Souza based on the holotype (*R. Romero & J. N. Nakajima 1039*).

##### Diagnosis.

The coriaceous leaf blade, broadly ovate to orbicular, entire and adpressed-margin, with 11–17 basal acrodromous veins, the stamens with a short pedoconnective and an inconspicuous ventral appendage together with the white petals, distinguish this species from another *Pleroma*.

**Figure 2.**
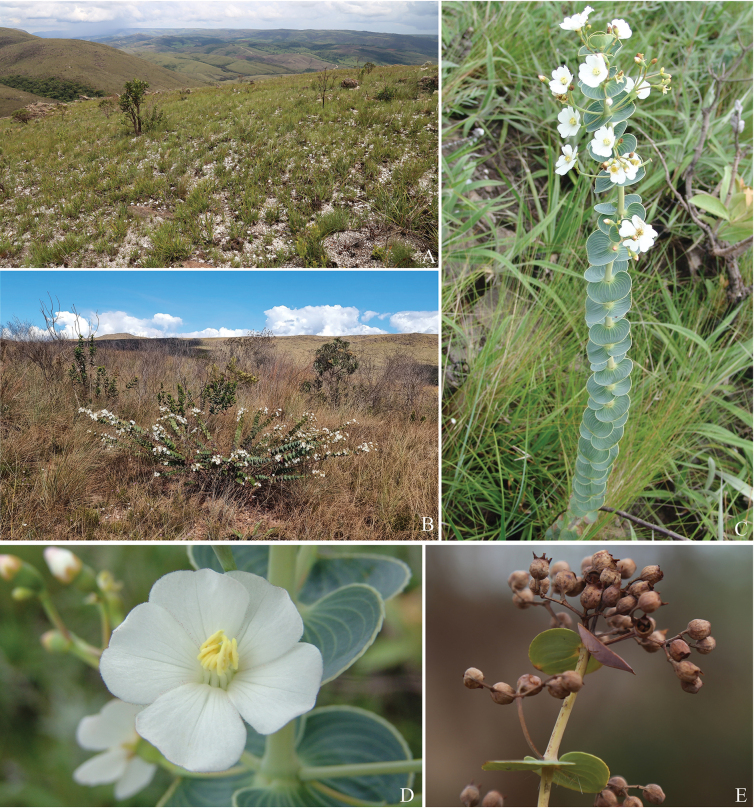
*Pleromacanastrense* R.Romero, R.Pereira & P.J.F.Guim. **A** rocky outcrop at Serra da Canastra National Park, Minas Gerais, Brazil, the type locality of *Pleromacanastrense***B** subshrub in campo rupestre near the road **C** flowering specimen showing the beautiful leaf architecture **D** flower **E** fruiting branch. Photos: Rosana Romero.

##### Description.

***Subshrubs*** or ***shrubs***, 0.6–1.8 m tall, decumbent. ***Stem*** terete to subterete, glabrous, or with setose trichomes in the basal portion. Younger branches quadrangular, flattened at the apex, with spherical glands, or glabrous, older branches terete, glabrous, internodes 2–5 cm long. ***Leaves*** sessile, decussate, horizontal, isomorphic in size at each node; blade 2.5–6.5 × 2–5 cm, discolorous (when fresh and dry), coriaceous, adaxial surface darker, broadly ovate to orbicular, obtuse to acute at the apex, rarely rounded, with a terminal trichome, 0.5–1 mm long, base cordate, semiamplexicaul, margin entire, adpressed-strigose, marginal trichomes 2–4 mm long, both surfaces glabrous, acrodromous veins 11–17, basal, principal vein lighter, conspicuously visible on both surfaces and secondary veins tenuous on the abaxial surface. ***Panicles*** of simple or compound dichasium, reduced or not, terminal; bracts two, 5–12.5 × 3–8.5 mm, caducous, leafy, sessile, coriaceous, largely ovate to orbicular, elliptic-lanceolate, obtuse to acute at the apex, base truncate, semiamplexicaul, margin strigose-ciliate, trichomes 0.3–1 mm long, both surfaces glabrous; bracteoles two, 3–7.5 × 2–6.7 mm, caducous, brownish, cucullate, membranaceous, both surfaces covered with spherical glands, margin glandular-ciliate, mainly at the apical portion. ***Flowers*** 5-merous; pedicel 1–2.5 mm long, glabrous; hypanthium 3–7 × 3–6 mm, green or glaucous, cylindrical, glabrous, furfuraceous; calyx tube 0.3 mm long; sepals 2–4 × 1.5–3 mm, green or glaucous, scarious, oblong, rounded at the apex, margin glandular-ciliate, furfuraceous; petals 8–14 × 9–12 mm, white, largely ovate, rounded or slightly retuse at the apex, base attenuate, margin entire, inconspicuously glandular-ciliate; androecium subisomorphic, stamens 10; filaments 4–7 mm long, white, filiform, glabrous or with sparse glandular trichomes in the basal portion; anthers 3–5 mm long, cream, oblong, straight, pore inclined ventrally, pedoconnective ca. 0.2 mm long, ventral appendage ca. 0.1 mm long, slightly bilobed, dorsal appendage ca. 0.1 mm long, lobed, glabrous; ovary 5-locular, half-inferior, free apical portion densely sericeous; style ca. 9 mm long, cream, filiform, slightly curved at the apex, glabrous, stigma truncate. ***Fruits*** loculicidal, capsules coated by persistent cupuliform hypanthium, 6–8.3 × 5–6.8 mm, brownish, oblong. ***Seeds*** ca. 0.5 × 0.3 mm, brown, numerous, cochleate, testa papillose.

##### Distribution and habitat.

**Figure 3.**
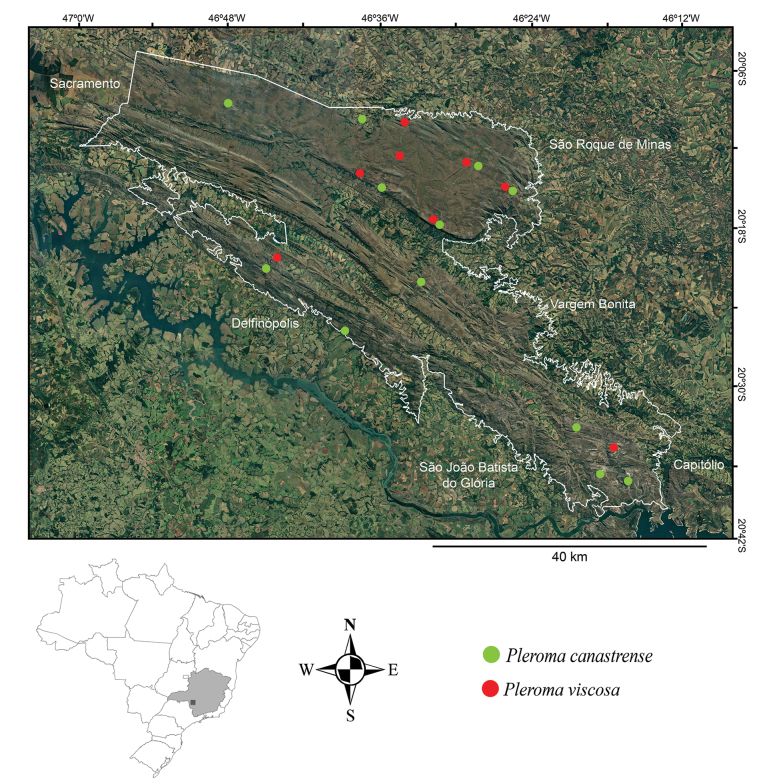
Geographical distribution of *Pleromacanastrense* and *Pleromaviscosa* in the Serra da Canastra National Park, Minas Gerais, Brazil [Adapted from Alves (2022)]. White line refers to the area of Serra da Canastra National Park.

So far, we know *Pleromacanastrense* only from the Serra da Canastra National Park, where it is probably endemic (Fig. 3). The beautiful populations with white flowers occur exclusively in campo rupestre.

##### Conservation status.

*Pleromacanastrense* has an area of occupancy (AOO) of 48 km^2^. Although this species has a distribution restricted to Serra da Canastra, we indicate it preliminarily here as “Least Concern” (LC), according to the IUCN categories (2012, 2022). This assessment is because most individuals are in a conservation unit with complete federal protection, established almost 50 years ago, with no significant threats to the populations. Furthermore, we have not identified a continued decline in occupancy over the past 30 years (R. Romero, pers. obs.).

##### Phenology.

*Pleromacanastrense* was collected in flower from April to August and in fruit from June to October.

##### Etymology.

The specific epithet refers to the restricted occurrence of this species in the campo rupestre of Serra da Canastra, Minas Gerais state, Brazil. The name “Serra da Canastra” is due to the similarity presented by the immense plateau, which, when viewed from afar, resembles a canastra, a term of Greek origin used to name a type of rustic chest with a rectangular shape.

##### Discussion.

*Pleromacanastrense* resembles *P.wurdackianum* (R.Romero & A.B.Martins) P.J.F.Guim. & Michelang., which also occurs in the Serra da Canastra National Park (Romero and Martins 2003). Both species share a subshrubby or shrubby habit, terete to subterete stem, glabrous and sometimes with setose trichomes on the basal portion, sessile leaves, discolorous leaf blade, cordate at the base, semiamplexicaul, and entire and adpressed-strigose margin with spiny trichomes. However, *P.wurdackianum* has pink petals (*vs.* white in *P.canastrense*), hypanthium and leaf blade with spherical glands (*vs.* glabrous). *Pleromacanastrense* is also similar to *P.integerrimum* (R.Romero & A.B.Martins) P.J.F.Guim. & Michelang., known only from Serra Preta in Delfinópolis (Romero and Martins 2003), since both have flowers with white petals. However, *P.integerrimum* differs in having a chartaceous and concolorous leaf blade (*vs.* coriaceous and discolorous in *P.canastrense*), glabrous at the margin (*vs.* adpressed-strigose).

Thirty years ago, John Julius Wurdack (1921–1998) wrote the following note on the sheet *Romero & Nakajima 1039* from HUFU herbarium: “*Svitramia* sp. *aff. S.hatschbachii* Wurdack (currently as *Pleromagertii* P.J.F.Guim. & Michelang.), but leaf cilia shorter, blade relatively broader and scarcely puncticulate, calyx lobes shorter, corolla white and larger, anthers longer.” *Pleromacanastrense* resembles *P.gertii*, the latter occurring preferentially in campo rupestre and more rarely in campo limpo and sujo in the south and southwest of Minas Gerais, *i.e.*, a distribution that exceeds the limits of the Serra da Canastra National Park. However, *P.gertii* has elliptic, elliptic-lanceolate, or ovate to ovate-lanceolate leaf blade (*vs.* broadly ovate to orbicular in *P.canastrense*) with 7–9 basal acrodromous veins (*vs.* 11–17), and purple petals (*vs.* white).

The specimen *G.J. Shepherd et al. 7032* (UEC055432) deposited at UEC was first named as *Svitramiaalba* by Angela Borges Martins (1945–) & João Semir (1937–2018) and this sheet annotated as holotype, but this name was never published by them.

##### Additional specimens examined (paratypes).

Brazil • Minas Gerais. Parque Nacional da Serra da Canastra. Capitólio: Reserva de Furnas; 20 February 1978; (fl), *G.J. Shepherd et al. 7032* (UEC055432-online image!) • Morro do Chapéu; 24 July 1993 (fl, fr); *R. Simão-Bianchini & S. Bianchini 413* (HUFU!, SP!) • Paraíso perdido, córrego Quebra-Anzol; 29 September 2005 (fr); *J.N. Nakajima 3737* (HUEFS!, HUFU!, VIC!) • estrada de Capitólio para Passos; 1 October 2005 (fl, fr); *R. Romero 7264* (HUFU!) • estrada para Pedreira Souza; 7 November 2005 (fl); *J.N. Nakajima et al. 4053* (HUFU!) • estrada atrás do Paraíso Perdido; 12 July 2006 (fr); *R. Romero et al. 7794* (HUFU!, RB!, SPF!, OUPR!, UEC!) • estrada para pedreira; 21 March 2007 (fr); *P.H.N. Bernardes et al. 45* (HUFU!, OUPR!, VIC!) • morro atrás da pousada do Rio Turvo; 21 May 2007 (fl, fr); *P.H.N. Bernardes et al. 140* (HUFU!, P!, UB!) • estrada para Cachoeira Feixo da Serra; 13 July 2007 (fl, fr); *R. Romero et al. 7795* (HUFU!, NY!, SPF!, RB!, UB!, UEC!, UFG!) • Chapadão de Furnas; 18 May 2013 (fr); *M.J.R. Rocha 985* (HUFU!, BHCB!) • Delfinópolis: estrada para a Babilônia; 24 May 1996 (fl, fr); *R. Romero & J.N. Nakajima 3438* (HUFU!) • Cachoeirinhas; 27 February 2000 (fr); *A.C.B. Silva 302* (HUFU!, SPF!, R!) • 2 May 2001 (fl, fr); *A.C.B. Silva 881* (HUFU!, SPF!, R!) • s.d. (fl, fr); *A.C.B. Silva 422* (HUFU!, SPF!, R!) • trilha Casinha Branca; 11 April 2002 (fl); *R.A. Pacheco et al. 101* (HUFU!) • trilha do Zé Carlinho, subida para a Serra do Cemitério; 9 October 2002 (fr); *J.N. Nakajima et al. 3193* (HUFU!, OUPR!, VIC!) • Paraíso Selvagem; 11 March 2003 (fl); *R. Romero et al. 6707* (HUFU!) • trilha do S. Cannyon; 16 May 2003 (fl, fr); *R. Romero et al. 6921* (HUFU!, UB!, UEC!) • estrada para Pedreira Souza; 30 September 2005; *J.N. Nakajima et al. 3946* (HUFU!, K!, OUPR!, P!) • estrada para Pedreira Souza; 17 February 2006; *R. Romero et al. 7704* (CESJ!, HUFU!, UFG!) • trilha Cachoeira Águas Claras; 23 June 2010 (fl); *P.O. Rosa et al. 1306* (HUFU!, K!, P!, VIC!) • São Roque de Minas: Parque Nacional da Serra da Canastra; 20 August 1994 (fl, fr); *R. Romero & J.N. Nakajima 1118* (HUFU!, SPF!, UFG!) • 15 October 1994 (fr); *J.N. Nakajima et al. 452* (HUFU!, OUPR!, UB!) • estrada São Roque de Minas para Sacramento; 16 October 1994 (fr); *R. Romero & J.N. Nakajima 1268* (HUFU!, VIC!) • Cachoeira Casca D’Anta; 17 October 1994 (fr); *J.N. Nakajima & R. Romero 554* (HUFU!, UEC!) • córrego dos Passageiros; 11 May 1995 (fl); *R. Romero et al. 2206* (CESJ!, HUFU!, NY!, OUPR!) • estrada para Cachoeira Casca D’Anta; 12 May 1995 (fl); *J.N. Nakajima et al. 1026* (HUEFS!, HUFU!, K!, P!, RB!, SPF!, UEC!, VIC!) • Parque Nacional da Serra da Canastra; 15 July 1995 (fl, fr); *R. Romero et al. 2384* (HUEFS!, HUFU!, OUPR!, RB!, SPF!, UEC!) • Cachoeira da Casca D’Anta; 17 July 1995 (fl, fr); *R. Romero et al. 2505* (CESJ!, HUFU!, NY!, OUPR!) • caminho para a Cachoeira Casca D’Anta; 18 July 1995 (fl, fr); *J.N. Nakajima et al. 1258* (HUEFS!, HUFU!, RB!, VIC!) • paredão da Cachoeira dos Rolinhos; 26 May 1996 (fl); *J.N. Nakajima & R. Romero 1775* (HUFU!, P!, UB!, UFG!) • Chapadão do Diamante; 9 July 1996 (fl); *J.N. Nakajima et al. 1929* (HUFU!, UEC!) • estrada para Cachoeira Casca D’Anta; 10 July 1996 (fl); J.*N. Nakajima et al. 1939* (HUFU!, UB!, UFG!) • 10 July 1996 (fl, fr); *J.N. Nakajima et al. 1947* (HUEFS!, HUFU!, K!, P!, US) • córrego dos Passageiros; 11 July 1996 (fl, fr); *J.N. Nakajima et al. 2011* (CESJ!, HUFU!, K!, NY!, US) • próximo à sede administrativa; 11 July 1996 (fl, fr); *J.N. Nakajima et al. 2043* (HUFU!, RB!, UEC!, VIC!) • Cachoeira Casca D’Anta; 10 August 1996 (fl); *J.N. Nakajima et al. 1948* (CESJ!, HUEFS!, HUFU!, OUPR!) • trilha do paredão da Serra da Canastra; 17 April 1997 (fl); *R. Romero et al. 4142* (HUFU!, SPF!, US!) • estrada para Fazenda do Fundão; 25 June 1997 (fl, fr); *R. Romero et al. 4265* (HUFU!, NY!, OUPR!, UEC!) • Cachoeira dos Rolinhos; 29 June 1997 (fl, fr); *R. Romero et al. 4369* (CESJ!, HUFU!, OUPR!) • Chapadão do Diamante; 29 June 1997 (fl); *R. Romero et al. 4330* (HUFU!, UB!, UFG!) (fl, fr), *R. Romero et al. 4336* (CESJ!, HUFU!, UFG!) • estrada para o sítio João Domingos; 20 August 1997 (fl); *J.N. Nakajima et al. 2635* (CESJ!, HUFU!, UB!, UFG!) • estrada para o Vale dos Cândidos; 22 August 1997 (fr); *J.N. Nakajima et al. 2708* (HUFU!, NY!, US!) (fr), *J.N. Nakajima et al. 2696* (HUFU!, RB!, UB!, VIC!) • Guarita de São Roque de Minas; 23 June 2001 (fl); *R. Romero et al. 6140* (CESJ!, HUFU!, UFG!) • parte alta da Cachoeira Casca D’Anta; 14 May 2007 (fl); *A.P.M. Santos & J.F. Silva 403* (HUEFS!, HUFU!) • morro próximo à sede administrativa; 23 July 2009; (fl), *P.J.F. Guimarães et al. 402* (RB!) • alto da Casca D’Anta; 29 May 2014 (fl); *A.F.A. Versiane et al. 647* (HUFU!, NY!).

#### ﻿ Pleroma viscosa

Taxon classificationPlantaeMyrtalesMelastomataceae

R.Romero, R.Pereira & P.J.F.Guim. sp. nov.

36E834AA-2D69-5DE0-BD05-101746058026

urn:lsid:ipni.org:names:77349020-1

Figs 3 , 4 , 5

##### Type.

Brazil • Minas Gerais. Parque Nacional da Serra da Canastra. São Roque de Minas: campo rupestre próximo à sede administrativa; 16 April 1994 (fl); *R. Romero et al. 845* (holotype: HUFU!; isotypes: BHCB!, RB!, UEC!, US!).

**Figure 4.**
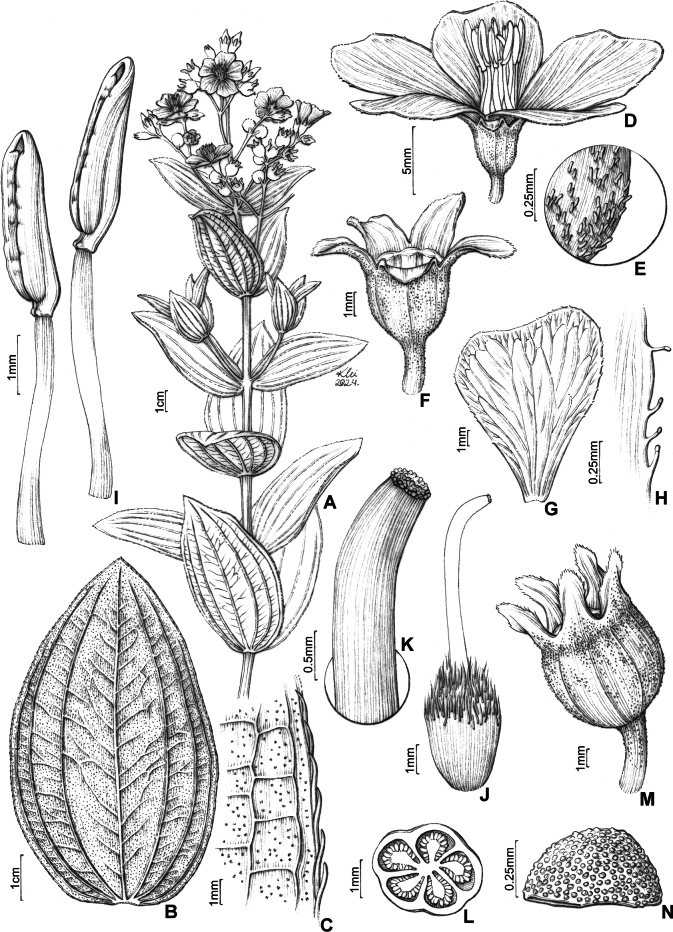
*Pleromaviscosa* R.Romero, R.Pereira & P.J.F.Guim.: **A** flowering branch **B** adaxial leaf surface **C** detail of the abaxial surface and margin of the leaf blade **D** flower **E** detail of the hypanthium surface **F** hypanthium and sepals **G** petal, abaxial view **H** petal margin detail **I** stamen, in lateral view **J** gynoecium **K** stigma detail **L** cross section of the ovary **M** fruit **N** seed (Illustration by Klei Souza based on the holotype (*R. Romero et al. 845*).

##### Diagnosis.

The viscosity in the younger branches, leaf blade, bracteoles, hypanthium, and sepals, due to the spherical glands, the prominent secondary veins on the abaxial surface of the blade, and the stamen with a very short pedoconnective (ca. 0.2 mm long), are characteristics that differ from those of other *Pleroma* species.

**Figure 5.**
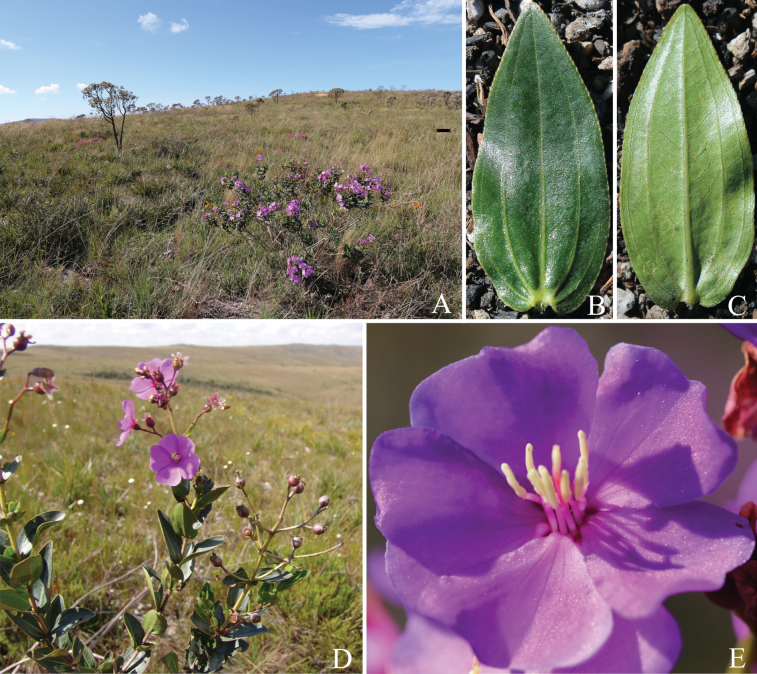
*Pleromaviscosa* R.Romero, R.Pereira & P.J.F.Guim., sp. nov. **A** flowering shrub in campo rupestre at Garagem de Pedras, Serra da Canastra National Park **B** adaxial leaf surface **C** abaxial leaf surface **D** flowering and fruiting branches **E** flower in frontal view. Photos: Rosana Romero.

##### Description.

***Subshrubs*** or ***shrubs***, 0.5–1.7 m tall, erect. ***Stem*** subquadrangular to quadrangular, sparse to densely covered with spherical glands, glutinous. Younger branches subquadrangular to quadrangular, flattened at the apex, with spherical glands, glutinous, older branches subquadrangular, with spherical glands, glutinous, internodes 2–5 cm long. ***Leaves*** sessile, decussate, horizontal, isomorphic in size at each node; blade 2–8 × 3–6 cm, discolorous (when fresh and dry), coriaceous, adaxial surface darker, ovate, ovate-oblong, elliptic-lanceolate to elliptic, obtuse or rounded at the apex, rarely acute, base rounded to subcordate, semiamplexicaul, margin entire, adpressed-strigose, marginal trichomes 1.5–2 mm long, both surfaces densely covered with brownish glands, glutinous, adaxial surface frequently glutinous, 5–7 acrodromous veins, basal, principal and secondary veins prominent on the abaxial surface. ***Panicles*** of simple or compound dichasium, reduced or not, terminal, densely covered with spherical glands, glutinous; bracts two, 2.5–9.5 × 4–8.5 mm, caducous, sessile, coriaceous, lanceolate or elliptic-lanceolate, obtuse or rounded at the apex, rarely acute, base truncate, semiamplexicaul, margin strigose-ciliate, trichomes 0.1–1.2 mm long, both surfaces densely covered with spherical, brownish glands, with glutinous aspect; bracteoles two, 4–5.5 × 3.5–4.3 mm, caducous, brownish, cucullate, membranaceous, both surfaces densely covered with spherical glands, with glutinous aspect, margin glandular-ciliate. ***Flowers*** 5-merous; pedicel 3.3–11 mm long, sparse to densely covered with spherical glands; hypanthium 3.3–5 × 3.5–4.5 mm, green or glaucous, cylindrical, with spherical glands; calyx tube 0.3 mm long, sepals 2–4.5 × 1.5–4 mm, green or glaucous, scarious, oblong, rounded at the apex, margin ciliate, rare sparse ciliate, with spherical glands and glutinous aspect; petals 11–17 × 11.5–16.5 mm, purple, obovate, rounded or slightly retuse at the apex, base attenuate, margin entire, glabrous or slightly ciliate; androecium subisomorphic, stamens 10; filaments 3.2–4.5 mm long, purple, filiform, glabrous, sometimes with sparse glandular trichomes; anthers 2.5–3.5 mm long, cream, oblong, slightly curved, pore inclined ventrally, pedoconnective ca. 0.2 mm long, ventral appendage ca. 0.1 mm long, slightly bilobed, dorsal appendage absent; ovary 5-locular, half-inferior, free apical portion densely sericeous; style ca. 8 mm long, cream, filiform, slightly curved at the apex, glabrous, stigma truncate. ***Fruits*** loculicidal, capsules coated by persistent cupuliform hypanthium, 6–8.5 × 5–6.8 mm, brownish, oblong. ***Seeds*** ca. 0.5 × 0.3 mm, brown, numerous, cochleate, testa papillose.

##### Distribution and habitat.

Like *P.canastrense*, *P.viscosa* is only known from Serra da Canastra National Park, where it is probably endemic (Fig. 3). Its populations also occur in campo rupestre, often associated with rocky outcrops.

##### Conservation status.

*Pleromaviscosa* has an area of occupancy (AOO) of 32 km^2^. Like *P.canastrense*, *P.viscosa* populations are restricted to Serra da Canastra National Park. Therefore, we propose, for the same reasons, the preliminary category of “Least Concern” (LC) according to IUCN (2012, 2022). We also did not identify a continuous decline in occupancy of *P.viscosa* populations over the past 30 years (R. Romero, personal obs.).

##### Phenology.

*Pleromaviscosa* was collected in flower from January to July and in fruit from April to October.

##### Etymology.

The specific epithet refers to the viscous appearance of the plant due to the spherical glands of the younger branches, on both sides of the leaf blade, bracteoles, hypanthium, and sepals. The substance produced by the spherical glands often stains newspapers where they are dried (Romero 2000).

##### Discussion.

*Pleromaviscosa* resembles *P.gertii* in having an erect stem, sessile leaves, coriaceous and discolorous leaf blade, with the adaxial surface darker than the abaxial surface. In addition, both species have a leaf blade obtuse to rounded at the apex, rounded to subcordate at the base, semiamplexicaul, with entire and adpressed-strigose margin. However, in *P.viscosa*, the leaf blade is covered with brownish glands (*vs.* yellowish green in *P.gertii*), which give a glutinous appearance mainly on the adaxial surface (*vs.* non-glutinous). Furthermore, the secondary veins are prominent on the abaxial surface of the leaf blade (*vs.* inconspicuous in *P.gertii*).

*Pleromaviscosa* also resembles *Pleromaminus* (R.Romero & A.B.Martins) P.J.F.Guim. & Michelang., also found in the Serra da Canastra National Park (see Romero and Martins 2003). Both species share coriaceous and discolorous leaf blade, entire and adpressed-strigose margin, both surfaces are covered with brownish spherical glands, and purple petals. However, *P.minor* can be distinguished by its glabrous or sparsely setose stem in the basal portion (*vs.* spherical glands in *P.viscosa*), ascending leaves (*vs.* horizontal), smaller leaf blade 0.8–4.5 × 0.5–2.5 cm (*vs.* 2–8 × 3–6 cm), pedicel ca. 2 mm long (*vs.* 3.3–11 mm long), petals rounded at the apex, and ciliate at the margin (*vs.* rounded or slightly retuse, glabrous or slightly ciliate).

The specimen *W.A. Araújo 11528* (ESA066062) deposited at ESA herbarium was first identified by Henrique Lahmeyer Mello Barreto (1892–1962) as *Svitramiawilson-araujaei* but this name was never published.

##### Additional specimens examined (paratypes).

Brazil • Minas Gerais Parque Nacional da Serra da Canastra. Araxá: Serra do Taquaral, divisas de Araxá e Sacramento; *W.A. Araújo* & *H.L. Mello Barreto 11528*; 25 May 1943 (ESA06606-image online!) • Delfinópolis: trilha Condomínio de Pedra; 23 June 2010 (fl); *R. Romero et al. 8258* (HUFU!, RB!) • São Roque de Minas: 20 February 1994 (fl); *R. Romero & J.N. Nakajima 625* (HUFU!, SPF!, UB!, UEC!) • 15 October 1994 (fr), *J.N. Nakajima et al. 469* (HUFU!, UB!, UEC!) • estrada para Sacramento; 17 October 1994 (fr); *J.N. Nakajima et al. 532* (CESJ!, HUFU!, UFG!) • 10 January1995 (fl); *R. Romero et al. 1642* (HUFU!, UFG!) • 12 January 1995 (fl); *R. Romero et al. 1779* (HUFU!, VIC!) • morro próximo à sede administrativa; 17 March 1995 (fl); *R. Romero et al. 1899* (HUFU!, K!, NY!, OUPR!, P!, US!) • estrada do Chapadão Diamante; 18 March 1995 (fl, fr); *J.N. Nakajima et al. 821* (CESJ!, HUFU!) • morro próximo à sede administrativa; 10 May 1995 (fl, fr); *J.N. Nakajima et al. 998* (HUFU! RB!, SPF!, VIC!) • 15 July 1995 (fl); *R. Romero et al. 2383* (HUFU!) • 11 January 1996 (fl, fr); *R. Romero et al. 3250* (CESJ!, HUFU!, UFG!) • Cachoeira Casca D’Anta; 13 January 1996 (fl, fr); *R. Romero et al. 3293* (HUFU!, K!, NY!, SPF!, UB!, UEC!) • morro após a nascente do Rio São Francisco; 20 March 1996 (fl); *R. Romero & J.N. Nakajima 3351* (HUFU!, K!, P), *R. Romero & J.N. Nakajima 3352* (HUEFS!, HUFU!, RB!, VIC!) • Serra Brava; 22 March 1996 (fl); *R. Romero & J.N. Nakajima 3393* (CESJ!, HUFU!, UFG!) • morro próximo à sede administrativa; 24 March 1996 (fl, fr); J.*N. Nakajima & R. Romero 1736* (HUFU!, K!, OUPR!, US!) • Chapadão do Diamante; 9 July 1996 (fl); *J.N. Nakajima et al. 1904* (CESJ!, HUFU!, OUPR!, UFG!) • morro próximo à sede administrativa; 16 April 1997 (fl); *R. Romero et al. 4063* (HUFU!, OUPR!, P) • Chapadão do Diamante; 18 April 1997 (fr); *J.N. Nakajima et al. 2318* (HUFU!, RB!) • morro próximo à sede administrativa; 9 January 1998 (fl); *R. Romero et al. 4832* (HUFU!, NY) • estrada a caminho da nascente do Rio São Francisco; 23 March 1999 (fl); *S.I. Elias & G.S. Rolim 341* (HUFU!) • Garagem de Pedras; 21 June 2001 (fl, fr); *R. Romero et al. 6136* (HUFU!, SPF!) • ca. de 2 km da sede; 23 June 2001 (fl); *R. Romero et al. 6141* (HUFU!, UB!, UEC!) • III.2007 (fl, fr); *C.M. Rodrigues 24* (HUEFS!, HUFU!) • morro próximo à sede administrativa; 23 July 2009, (fr); *P.J.F. Guimarães et al. 400* (RB!).

### ﻿The Melastomateae tribe from the Serra da Canastra National Park

Of the 24 species of Melastomateae cataloged for the Serra da Canastra National Park, nine are endemic to the Park and its surroundings: *Pleromabergianum* (Cogn.) P.J.F.Guim. & Michelang., *P.canastrense* R.Romero, R.Pereira & P.J.F.Guim., *P.gertii* P.J.F.Guim. & Michelang., *P.integerrimum* (R.Romero & A.B.Martins) P.J.F.Guim. & Michelang., *P.minus* (R.Romero & A.B.Martins) P.J.F.Guim. & Michelang., *P.petiolatum* (R.Romero & A.B.Martins) P.J.F.Guim. & Michelang., *P.rubrobracteatum* (R.Romero & P.J.F.Guim.) P.J.F.Guim. & Michelang., *P.viscosa* R.Romero, R.Pereira & P.J.F.Guim., and *P.wurdackianum* (R.Romero & A.B.Martins) P.J.F.Guim. & Michelang. *Pleromabandeirae* P.J.F.Guim. & Michelang. also occurs in the southern portion of Minas Gerais, in Lavras, Carrancas, São Tomé das Letras, Tiradentes, and São João Del Rey. *Chaetogastraminor* (Cogn.) P.J.F.Guim. & Michelang. occurs in São Paulo, Minas Gerais, and Rio de Janeiro (Goldenberg et al. 2024). The other species have a wide distribution in Brazil: *Chaetogastragracilis* (Bonpl.) DC., *C.herbacea* (DC.) P.J.F.Guim. & Michelang., *Macairearadula* (Bonpl.) DC., *Pleromaheteromallum* (D.Don) D.Don, *P.martiale* (Cham.) Triana, *P.stenocarpum* (Schrank et Mart. ex DC.) Triana, *P.candolleanum* (Mart. ex DC.) Triana, *P.estrellense* (Raddi) P.J.F.Guim. & Michelang., *P.fothergillii* (Schrank et Mat. ex DC.) Triana, P. *frigidulum* (Schrank et Mart. ex DC.) Triana, *P.oleifolium* (DC.) R.Romero & Versiane, *Pterolepisrepanda* (DC.) Triana, and *Tibouchinaaegopogon* (Naudin) Cogn.

The species of the Melastomateae tribe from the Serra da Canastra National Park, Minas Gerais, Brazil can be identified by the key below.

### ﻿Key to the species of the Melastomateae tribe from the Serra da Canastra National Park, Minas Gerais, Brazil

**Table:** 

1	Branches, hypanthium, leaves, bracts, and sepals with indumentum lepidote	***Tibouchinaaegopogon***
–	Branches, hypanthium, leaves, bracts, and sepals with indumentum non-lepidote	**2**
2	Flowers 4-merous	**3**
–	Flowers 5-merous	**5**
3	Herbs; hypanthium with penicellate emergences	***Pterolepisrepanda***
–	Subshrubs or shrubs; hypanthium without penicellate emergences	**4**
4	Branches densely strigose; petals pink with a cream base	***Macairearadula***
–	Branches densely glandulous; petals entirely purple	***Chaetogastraherbacea***
5	Herbs or subshrubs, unbranched	**6**
–	Shrubs or subshrubs branched and/or trees	**7**
6	Herbs stoloniferous, 0.1–0.3 m tall; leaf blade 0.1–2.4 × 0.1–1.4 cm	***Chaetogastraminor***
–	Subshrubs non-stoloniferous, 0.4–0.8 m tall; leaf blade 2.5–9 × 1.5–4 cm	***Chaetogastragracilis***
7	Pedoconnective with ventral appendage glandulous	**8**
–	Pedoconnective with ventral appendage glabrous	**10**
8	Hypanthium densely sericeous; petals purple with white base turning reddish	***Pleromaheteromallum***
–	Hypanthium sericeous-stellate or setose; petals entirely purple	**9**
9	Hypanthium sericeous-stellate; panicles 6–12 cm long	***Pleromacandolleanum***
–	Hypanthium setose; panicles 1.5–5 cm long	***Pleromafothergillii***
10	Leaf blade and bracts dendritic-stellate or sericeous-glandular	**11**
–	Leaf blade and bracts strigose, bullate-strigose, sericeous-stellate, adpressed-strigose, or sericeous	**12**
11	Hypanthium sericeous-glandulous; bracteoles 11–12.5 × 10–13 mm, reddish	***Pleromarubrobracteatum***
–	Hypanthium dendritic-stellate; bracteoles 5–6.5 × 2–3.5 mm, cream	***Pleromaoleifolium***
12	Filaments setose or glabrous	***Pleromamartiale***
–	Filaments glandular	**13**
13	Subshrubs or shrubs; branches non-winged	**14**
–	Trees; branches winged or sub-winged	**15**
14	Scales between the sepals present; verticillate leaves	***Pleromafrigidulum***
–	Scales between the sepals absent; decussate leaves	**16**
15	Adaxial surface of the leaf blade strigose, abaxial surface sericeous; hypanthium sericeous	***Pleromastenocarpum***
–	Adaxial surface of the leaf blade bullate-strigose, abaxial surface sericeous-stellate; hypanthium strigose-stellate	***Pleromaestrellense***
16	Leaf with a long petiole 5–10 mm long	***Pleromapetiolatum***
–	Leaf with a short petiole 1–5 mm long or sessile	**17**
17	Style with trichomes at the base	***Pleromabergianum***
–	Style glabrous	**18**
18	Leaf blade chartaceous, concolorous; margin glabrous	***Pleromaintegerrimum***
–	Leaf blade coriaceous, discolorous; margin entire, adpressed-strigose	**19**
19	Leaf blade glabrous, except for the margin	**20**
–	Leaf blade with indumentum	**21**
20	Petals purple	***Pleromawurdackianum***
–	Petals white	***Pleromacanastrense***
21	Both surfaces of the leaf blade with brownish glands	**22**
–	Both surfaces of the leaf blade with yellowish-green glands	**23**
22	Stem glabrous or sparsely setose; leaves ascending, blade 0.8–4.5 × 0.5–2.5 cm, leaf marginal trichome 0.8–1.5 mm long	***Pleromaminus***
–	Stem covered with spherical glands; leaves horizontal, blade 2–8 × 3–6 cm, leaf marginal trichome 1.5–2 mm long	***Pleromaviscosa***
23	Branches and leaf blade with setose-adpressed trichomes; hypanthium sericeous	***Pleromabandeirae***
–	Branches glabrous or with spherical glands; leaf blade with spherical glands; hypanthium glabrous	***Pleromagertii***

## Supplementary Material

XML Treatment for Pleroma canastrense

XML Treatment for Pleroma viscosa

## ﻿Additional information

### Author ORCIDs

Rosana Romero https://orcid.org/0000-0003-1090-7557

Rodrigo Pereira Silva https://orcid.org/0000-0002-7969-6324

Paulo José Fernandes Guimarães https://orcid.org/0000-0001-5266-4552

### Data availability

All of the data that support the findings of this study are available in the main text.
